# The perceptions of nurse managers working in the mining industry on workplace relationships

**DOI:** 10.4102/hsag.v28i0.2120

**Published:** 2023-08-29

**Authors:** Sanele E. Nene

**Affiliations:** 1 Department of Nursing, Faculty of Health Sciences, University of Johannesburg, Johannesburg, South Africa

**Keywords:** perceptions, primary healthcare, nurse managers, workplace, relationships

## Abstract

**Background:**

Positive workplace relationships improve the quality of healthcare provided by promoting teamwork and ensuring that challenges are addressed amicably. Nurse managers, unit managers, nurses, doctors and union leaders are obligated to establish positive working relationships to deliver quality primary healthcare service to mine workers at the clinics of the mine. However, there are negative workplace relationships at the clinics of a specific mining company that affect the quality of service delivered.

**Aim:**

The purpose of this study was to explore and describe the perceptions of primary healthcare nurse managers working in the mining industry on workplace relationships.

**Setting:**

This study was conducted in mining primary healthcare clinics located in Gauteng province, in the West Rand.

**Methods:**

A qualitative, exploratory, descriptive and contextual research design was adopted in this study. Individual interviews were conducted to collect data from the primary healthcare nurse managers, and data were analysed using thematic analysis. Trustworthiness measures and ethical principles were upheld in this study.

**Results:**

Three themes surfaced in this study: (1) negative perceptions of primary healthcare nurse managers on workplace relationships, (2) positive perceptions of primary healthcare nurse managers on workplace relationships and (3) building workplace relationships between primary healthcare nurse managers and union leaders.

**Conclusion:**

Nurse managers had positive and negative perceptions on working relationships in this study. Negative workplace relationships should be prevented at all costs by resolving conflicts amicably.

**Contribution:**

Resolving of conflicts amicably to build positive workplace relationships.

## Background

A workplace perceived by both employers and employees as being healthy or positive is of utmost importance (Ntimba 2019). According to Persson et al. (2018), such workplace environments retain their employees and increase their commitment towards the achievement of organisational goals. Positive workplace relationships in healthcare create positive psychosocial work environment that results in the achievement of positive health outcomes (Dickson-Swift et al. 2014; Sweeney & Swan 2014; Thylefors 2013).

Nurses and unit managers are reporting to the nurse managers. The working relationships among the three stakeholders largely influence their key performance indicators and job satisfaction (McCay, Lyles & Larkey 2018; Nene 2021). Nurmeksela et al. (2021) argue that nurse managers’ work affects nursing outcomes in complex ways. Squires et al. (2017) elicit that nurse managers should not just ensure that unit managers and nurses have adequate resources to perform their tasks, but they should also foster positive working relationships among nurses. Lotfi et al. (2018) concluded that where positive workplace relationships are not encouraged and advocated for, patients suffer the consequences of poor healthcare service delivery.

Doctors are expected to manage conditions of mining primary healthcare patients that cannot be managed by the registered nurses or primary healthcare nurse practitioners. Nurses and doctors work collaboratively to heal the patients (World Health Organization 2016). El Sayed and Sleem (2011) and Mahboube et al. (2019) posit that positive working relationships between nurses and doctors is considered the main factor in achieving positive medical results, which is the ultimate goal of the healthcare system. Hoseini (2009) and Nene (2022) attest that nurses and doctors should effectively and efficiently establish communication and believe that neither can achieve the desirable goal alone; in other words, they should take necessary steps towards creating positive workplace relationships to promote teamwork.

In mining primary healthcare clinics, positive workplace relationships are essential to influence the quality of service rendered positively and the well-being of employees (Haar et al. 2019). Union leaders are a critical stakeholder in mining primary healthcare clinics, and their obligation is to protect the healthcare rights of the mine workers and to advocate for them to receive quality healthcare service (Shen, Chou & Schaubroeck 2019; Wohlgemuth 2017). Nurse managers and union leaders should establish positive workplace relationships between them to ensure that the primary healthcare-related complaints and challenges are addressed amicably (Nene, Ally & Nkosi 2020). Positive workplace relationships have a potential to allow nurse managers and unions to even discuss their personal challenges, as outside work issues do affect work if they are not addressed (Mind Tools 2021). Wohlgemuth (2017) added that the work pressures do not cause employees strain or anxiety, emotional exhaustion and a lack of fulfilment, when positive workplace relationships are existing within their organisations. Studies on workplace relationships revealed that positive working relationships are fundamental to getting things done and achieve excellent results (Darley & Latané 1970; Gouldner 1954; Persson et al. 2018).

*South African Government, Mine Health and Safety Act* (1996) and World Health Organization (2016) prescribe, monitor and advocate for good working conditions and ensuring safety in the workplace, but they are overlooking the need for establishment of positive workplace relationships among the nurses, doctors and nurse managers and the union leaders. This is confirmed by Persson et al. (2018) who articulate that there is a need for a better understanding of what generates positive working relationships, as efforts to improve these relationships could increase the general quality of the workplace climate. The more comfortable the employees are around one other, the more confident they will feel voicing opinions, brainstorming and going along with new ideas, and when they see the successes of working together in this way, group morale and productivity will soar (Mind Tools 2021).

## Problem statement

In 8 out of 10 mines, there is poor workplace relationship in South Africa (Haar et al. 2019; Ntimba 2019; Persson et al. 2018). Hence the researcher’s observations are that the mine workers, union leaders and the mining management seem to be pulling in the opposite directions. This poor workplace relationship replicates to the mining primary healthcare clinics where the poor workplace relationship is evident among staff, and this affects the quality of service rendered at these clinics. According to Shezi (2015) and Nene et al. (2020), the poor workplace relationship in South African mines is characterised by a lack of respect, disrespect of boundaries, negative attitudes towards each other, and poor communication. Bendix (2019) suggests that challenges and conflicts in the workplace should not be wished away but should be properly managed and in a constructive manner to yield positive results. If negative workplace relationships are not resolved, the working environment will be unconducive for employees to thrive, the production will drop, targets will not be met, and it will also increase the employee turnover (Ntimba 2019).

The following research question surfaced from the background and the problem statement:

What are the perceptions of primary healthcare nurse managers working in the mining industry in Gauteng on workplace relationships?

## Purpose of the study

The purpose of this study was to explore and describe the perceptions of primary healthcare nurse managers working in the mining industry on workplace relationships.

### Setting

This study was conducted in a mine, located in Gauteng, in the West Rand. In this mine, 15 primary healthcare nurse managers are leaders of more than eight onsite primary healthcare clinics, with 45 nursing personnel reporting to them, and four medical doctors providing services to 17 000 mine workers (Nene 2021; Nene et al. 2020). A majority of these clinics operate for 8 h and some operate on a 24-h basis. The primary healthcare nurse managers oversee all the activities happening in the clinics. Each clinic has the unit manager that report to the primary healthcare nurse managers. These primary healthcare clinics are not outsourced but are managed by the mine and are rendering comprehensive primary healthcare services such as emergency services that are at primary healthcare level, medical and surgical services, maternal care and women’s health, health promotion and management of chronic diseases.

The mine workers that these clinics are providing services to are all affiliated with a union of their choice, which is led by union leaders. One of the nurse managers’ leadership roles was to address the primary healthcare-related complaints raised by the union leaders, and these complaints included: (1) long waiting times, (2) sending an employee back to work too early and (3) not booking the employee off sick, to count the few (Nene et al. 2020).

## Research methods and design

A qualitative, exploratory, descriptive and contextual research design and a constructivist paradigm were adopted in this study. This design and paradigm assisted the researcher to describe and explore the perceptions of primary healthcare nurse managers working in the mining industry on workplace relationships.

### Population and sample

The participants of this study were primary healthcare nurse managers working in a mining industry, employed by the mining company. Of 15 primary healthcare nurse managers who formed the population of the study, only 10 of them met the selection criteria and became the sample of the study through purposive sampling. The selection criteria were being employed in mining primary healthcare clinics for more than a year, registered with the South African Nursing Council as nurse administrators and being available for interviews during the data collection period, which was from 01 September 2017 to 18 December 2017. The participants were recruited in an information session meeting that the researcher held with them prior to signing of the consent forms to participate in the study. There was no gatekeeper used to access the participants in this study; the researcher contacted the participants directly.

### Data collection

Data were collected from primary healthcare nurse managers, by the researcher using semi-structured in-depth individual interviews, in their offices in the workplace. Permission to conduct the study was obtained from the Director of Health and Wellness of the mines, and the researcher was permitted to contact the participants directly. The researcher sent e-mails inviting the participants to an informative session meeting. After the meeting, nurse managers who demonstrated interest to participate in the study granted their consent in writing. The dates, times and venues of the interviews were arranged to suit the schedule of the nurse managers and each interview lasted not more than 45 min. The following open-ended question was posed to the participants during the semi-structured interviews: ‘What are your perceptions on workplace relationships in this mining company?’ This question was followed by probing, which was according to the responses of the participants. Field notes were also collected, typed up and added to the transcripts to attach and describe the gestures and emotions expressed by the participants during data collection. No new information surfaced by the seventh participant, meaning data were saturated; three more interviews were conducted to confirm data saturation. All interviews were audio-recorded to improve the credibility of data collected and consent was received from the participants.

### Data analysis

Giorgi’s descriptive method of data analysis was used to analyse data (Holloway & Gavin 2017). The researcher and an independent coder coded the data independently, grouping of transcripts was done, and themes were formed using simple descriptive words. They then held a consensus meeting to discuss the themes that emerged.

### Trustworthiness

Four strategies of trustworthiness as outlined by Lincoln and Guba (1985) were applied to ensure the trustworthiness of the study. These strategies include credibility, dependability, transferability and confirmability. Credibility was maintained by using more than one method to collect data: individual interviews and field notes. Audio-recordings and verbatim transcriptions of interviews also assisted to ensure the credibility of this study. The findings of the study were confirmed by an independent coder to ensure its dependability and were also presented to the participants and the management of the mining company. A detailed description of the research method and study setting ensured that the findings of this study can be transferrable to other similar settings. The research process was closely monitored by the researcher’s supervisors and regular meetings were held to clarify, correct and reach consensus; this ensured confirmability of the study findings.

### Ethical considerations

The principles of autonomy, non-maleficence, beneficence and justice are the ethical principles that were upheld throughout the study. Ethical clearance was obtained from the University of Johannesburg Faculty of Health Research Ethics (REC 01-73-2017). The informed consents to participate in the study and for audio-recording were signed by all the participants. Participants participated voluntarily, and their right to withdraw from the study any time before the completion of data analysis was upheld. At the time of data collection, the researcher was employed as a Case Manager at the mining primary healthcare clinics reporting to the Group Case Manager and did not have any close relationship with the participants. No harm was imposed on the participants during this study. Privacy and confidentiality were maintained throughout the study, by not disclosing the personal information of the participants at any stage of the study and by keeping the raw data of the study in a locked safe at a home of a researcher. Raw data that was kept in the laptop of the researcher were security protected. Only the researcher, the supervisors and the independent coder had access to the raw data of this study.

## Results

Three themes and a number of subthemes surfaced in this study. The themes were: (1) negative perceptions of primary healthcare nurse managers on workplace relationships, (2) positive perceptions of primary healthcare nurse managers on workplace relationships and (3) building workplace relationships between primary healthcare nurse managers and union leaders. The summary of the participants’ demographics is provided in Table 1. Ten participants interviewed consisted of nine black people and one white person, four males and six females. Four of the participants have Diplomas in Nursing, Midwifery and Nursing Management, while five of them have a BCur Educationis Et Admin degree and one has a BCur Educationis Et Admin degree and a Master’s degree. Their age ranged between 38 and 60 years and were all registered with the South African Nursing Council as nurse administrators.

**TABLE 1:** Participants’ demographics.

Variables	Number
**Race**	
Black people	9
White people	1
**Gender**	
Male	4
Female	6
**Highest qualifications:**	
Diploma(s)	4
BCur Educationis Et Admin degree	5
BCur Educationis Et Admin degree and Master’s degree	1
Age range (years)	38–60
Registered with the South African Nursing Council	10

### Theme 1: Negative perceptions of primary healthcare nurse managers on workplace relationships

Nurse managers had negative perceptions only when it comes to union leaders; they alluded that union leaders are autocratic, difficult and become a challenge when they expect them to be team members. A communication barrier and a lack of teamwork between nurse managers and union leaders were mentioned as negative perceptions as well. Below are some of the verbatim quotations from the participants:

‘We are working in a highly unionised environment; our unions are very autocratic, whatever they say it must work, they always want to be involved even in confidential matters that are for nurses and patients only.’ [*looking worried*] (P1, Male, 45 years)

‘It is very difficult for us to engage with the union leaders without a fight really, even in simple and straight forward things.’ (P6, Female, 43 years)

‘Union leaders are a challenging stakeholder for us, you will be expecting them to be team members, support you with one, two and three, but they won’t.’ [*taking a deep breath*] (P3, Female, 57 years).

Negative perceptions of primary healthcare nurse managers on workplace relationships were mentioned by the participants as autocratic union leaders, difficult union leaders and communication barrier and a lack of teamwork between nurse managers and union leaders.

#### Autocratic union leaders

The participants mentioned that the union leaders are autocratic, and this is affirmed by the following quotations from the participants.

‘Look to be honest with you, our unions are very demanding, you get that yes it is your responsibility to give them information about certain issues happening in our primary healthcare clinics but the way they ask that information is not nice, they can be very aggressive.’ (P10, Female, 52 years)

‘You know at times union leaders will be telling you that, “you must do what we tell you do, stop asking us questions the mine workers are our [*union leaders*’] members.” The you ask yourself to say but these leaders do they understand that this is not ethical.’ (P3, Female, 57 years)

#### Difficult union leaders

Other participants perceived that union leaders are difficult to deal with; confirming this are the following quotations from the participants:

‘Unions are very challenging and difficult to work with I am telling you, you will hear them saying I don’t agree with one, two and three. Then you end up asking yourself, what is it that they are agreeing with on what we are saying and you get that they are not agreeing with anything you are saying.’ (P2, Female, 50 years)

Using both hands to explain and looking worried:

‘The union leaders are always ready to fight, they always want to push you to the edge, even if you are not in the mood, these people are very difficult to be honest, they fight over nothing … nothing.’ [*looking angry*] (P1, Male, 45 years)

#### Communication barrier and lack of teamwork between nurse managers and union leaders

The participants reported that there is a communication barrier and a lack of teamwork between nurses and union leaders in this study; supporting this statement are the following quotations uttered by the participants:

‘There is always a communication barrier between us and unions, you get that our senior management is telling us this and the unions are talking a different story and at times we end up confused to say what exactly must we perceive as true.’ (P5, Female, 40 years)

‘At times we will be inviting unions to come on board to discuss certain matters of our patients who are their members, then they will be refusing completely or you get that we discuss a matter and agree that this will be a way forward then few days later the unions have changed their minds and when they do that they don’t come back to you to say no things have changed.’ [*looking concerned*] (P4, Male, 38 years)

From the findings presented above, it can be concluded that primary healthcare nurse managers find it difficult to work with the union leaders because these leaders are aggressive at times and want to tell the nurse managers what to do. Another negative perception that emanated from the findings was a communication barrier between the primary healthcare nurse managers and union leaders, which ends up confusing nurse managers what to perceive as true, and a lack of teamwork between these two role players of primary healthcare in the mining industry.

### Theme 2: Positive perceptions of primary healthcare nurse managers on workplace relationships

Positive perceptions on workplace relationships were mentioned by the participants as learning from each other, unity and good team spirit among the nurses and doctors at the clinics. This is supported by the following verbatim quotations from the participants:

‘Let me tell you, our nurses and doctors are learning from one another, remember doctors scope is broader than ours as nurses, so our doctors will be using every chance they get to teach our nurses, and they will be asking how they nurse such conditions and our nurses will be responding.’ [*smiling*] (P9, Male, 48 years)

‘What is good is that we do not allow anything or anyone to come between us as nurses and doctors, we are here for the patients, we work as a team and remain united.’ (P2, Female, 50 years)

‘I really think we have a very good working relationship between our nurses and doctors really, they are always there for each other, they are united, no matter how busy the clinics are, they work as a team.’ [*looking excited*] (P6, Female, 43 years)

The positive perceptions of the participants on working relationships surfaced as unity and positive team spirit among the primary healthcare nurses and unity and positive team spirit between nurses and doctors.

#### Unity and positive team spirit among the primary healthcare nurses

The participants alluded that there is unity and team spirit among nurses in these primary healthcare clinics and this is confirmed by the following verbatim quotations:

‘You know there is a collective amongst our nurses, they share knowledge and expertise, they work together and are very united.’ (P6, Female, 43 years)

‘When nurse A is trained on a certain component, nurse A will ensure that they share their knowledge or the information with the other nurses who are not yet trained on that component. Our nurses know their weaknesses, they stand together and support one another, they want to win as a team and not as individuals.’ (P7, Male, 59 years)

#### Unity and positive team spirit between nurses and doctors

Unity and positive team spirit were mentioned by the participants in this study, and this is supported by the quotation below from one of the participants:

‘In other places you will find conflicts and poor team spirit between nurses and doctors, not with us. Our nurses and doctors are a team and this have a great influence on the quality of care we provide to our patients.’ (P3, Female, 57 years)

Another participant added:

‘It is exciting to see our nurses and doctors united and working together you know, they know they can call each other any time, you find them together even during tea time imagine.’ [*smiling and looking excited*] (P5, Female, 40 years)

The participants perceived the working relationship among nurses and between nurses and doctors as positive. According to these perceptions, the nurses and doctors are united and working as a team in these primary healthcare clinics of a mining company.

### Theme 3: Building positive workplace relationships between primary healthcare nurse managers and union leaders

The participants stressed the importance of building positive workplace relationships to promote teamwork. This is confirmed by the following quotations from the participants:

‘We need to build positive working relationships with the union leaders as a team, because this thing of them seeing us as enemies has to come to an end.’ (P2, Female, 50 years)

‘We will build these positive working relationships by dealing with the grievances directly as a team, and also by not fighting over nothing with the union leaders, as it happens.’ [*looking concerned*] (P1, Male, 45 years)

Another participant added that working relationships should be built by removing the communication walls between the nurse managers and unions leaders:

‘By removing the walls between union leaders and us we will be building positive working relationships with them and we have to ensure that we don’t dent that relationship as it will promote engagements and improve the quality of services we offer to mine workers.’ [*Using both hands to explain and smiling while talking*] (P8, Female, 46 years).

The building of positive workplace relationships between primary healthcare nurse managers and union leaders was mentioned as a root cause of challenges separating the nurse managers and union leaders, collective management of primary healthcare challenges and ensuring effective communication between these two parties.

#### Root cause analysis of challenges dividing the primary healthcare nurse managers and union leaders

The participants stated that they need to identify the challenges that are dividing them from the union leaders, and this is supported by the following verbatim quotations:

‘We need to find ways to identify the challenges that make it difficult for us to work together with the unions, we need to find a way not to dent the working relationship between us and these unions.’ (P1, Male, 45 years)

‘Us and unions need to seat in one room and say okay this is not working, why is it not working? And how can we make it work, then we will get somewhere.’ (P6, Female, 43 years)

#### Collective management of primary healthcare challenges

The participants also mentioned that they need to manage the primary healthcare challenges as a team. This statement is supported by the following quotations from the participants:

‘This thing of fights between us and union leaders is not helping, we need to address the primary healthcare grievances and challenges together, we are a team and should work as such.’ (P3, Female, 57 years)

‘I really cannot wait for days where union leaders pick up a phone and call us to ask about a certain case or come to us in a polite and professional manner and say here is the matter how can we address it as a team, or can you advise us on this matter and all that.’ (P2, Female, 50 years)

#### Ensuring effective communication between the primary healthcare nurse managers and union leaders

The participants also expressed that they need to ensure that there is effective communication between them and union leaders, the participants said:

‘We need to engage and involve union leaders as much as possible, especially in matters that affects them directly such as in employees who has a potential to me medically boarded because these employees first report in our primary healthcare clinics before they are referred to the occupational health centre.’ (P4, Male, 38 years)

In support of this another participant said:

‘An open and honest communication between us and unions is needed, a more structured communication system, we must end this thing of fighting over nothing, we need regular meetings as well to keep our communication channels clear as well.’ [*smiling and looking confident*] (P10, Female, 52 years)

It is essential to build positive workplace relationships between the primary healthcare nurse managers and union leaders. It surfaced from this study that this should be done by analysing the causes of challenges, managing these challenges as a team and by ensuring that there is effective communication between all parties involved.

## Discussion

It emanated in this study that the participants have negative perceptions, namely autocratic, difficult and challenging union leaders on workplace relationships. A communication barrier and a lack of teamwork between nurse managers and union leaders were also posited as negative perceptions by the participants. Ehlers (2017) reveals that employees perceive workplace relationships as negative when they are characterised by undesirable leaders who bully and victimise their subordinates and who are intentionally difficult to work with. Studies on workplace relationships reveal that negative workplace relationships affect the team interaction and performance, as well as the commitment of the employees towards the organisation (Duan et al. 2018; Harms et al. 2018; Shen et al. 2019). Conflicts between the nurse managers and unions are expected and should be resolved amicably to prevent negative workplace relationships that may result to poor employee performance (Ntimba 2019).

Ehlers (2017) opines that a lack of trust between managers and employees triggers negative perceptions on workplace relationships and employees exposed to negative workplace environment respond negatively to the abusive leaders’ behaviour by eliciting counter-behaviours that can be to the detriment of the organisation. Venter and Levy (2014) argue that the relationship itself, wage dispute, scarcity of resources and goal incongruence enforce unions to be autocratic, difficult and challenging to managers. Smith and Diedericks (2016) concluded that communication issues and a lack of teamwork between the union leaders and managers deny these two parties an opportunity to learn and grow from each other. Ntimba (2019) stated that unfortunately, the workplace is still characterised by antagonistic relations between managers and unions leaders who represent the employees in unionised work organisations. Nurse managers and union leaders should be working as a team and not against each other as this changes the negative perceptions that the nurse managers have on workplace relationships.

It surfaced in this study that the participants also have positive perceptions on workplace relationships, and these are unity and positive team spirit among the primary healthcare nurses and unity and positive team spirit between nurses and doctors. According to Persson et al. (2018), positive workplace relationships are a resource for reaching the goal of doing a good job. Jenny et al. (2017) concur that positive workplace relationships are important to the psychosocial work environment and may be resources for the employees’ well-being; there is, however, a need for a better understanding of what generates positive workplace relationships. Smith and Diedericks (2016) posit that where there is a prevalence of positive workplace relationships, positive employment relations are bound to happen. These authors added that effective communication, fairness, cohesion, satisfaction, commitment, productivity and goal attainment are outcomes of positive workplace relationships.

Ehlers (2017) asserted that nurse managers should create a positive workplace environment where nurses and doctors are able to learn from each other, work in unity and as a team to enhance their job satisfaction and organisational trust. This notion is supported by Ntimba (2019) and Wohlgemuth (2017) who postulated that unity and good team spirit between nurses and doctors enable healthcare organisations to achieve their strategic and operational goals. Positive perceptions of nurse managers on workplace relationships shows that there is a positive working environment in these primary healthcare clinics in a mining industry.

Participants mentioned that there is a need of building of positive workplace relationships between primary healthcare nurse managers and union leaders through root cause analysis of challenges dividing these two parties, collective management of primary healthcare challenges and ensuring effective communication. Smith and Diedericks (2016) attest that positive workplace relationships are built over time and develop into trusting, loyal and mutual connections that promote teamwork. Nene (2021) alluded that building positive workplace relationships in primary healthcare clinics requires identification of the causes of challenges a collective fusion to directly tackle the challenges encountered. According to the World Health Organization, Monitoring Health for Sustainable Development Goals (SDGs) (2019) posit that it is impossible to achieve collective goals and promote teamwork without building positive working relationships.

Kgatle (2018) stresses that union leaders and nurse managers are expected to lead by example; they should manage conflicts constructively and build positive working relationships that will benefit the mine workers they are serving. Persson et al. (2018) argue that positive workplace relationships promote teamwork; it is therefore essential to build these relationships from back where the damage of the relationships started. Nurse managers, nurses, doctors as well as union leaders should foster therapeutic discussions that will mould their working relationships and yield positive outcomes. Ehlers (2017) added that primary healthcare nurse managers have an obligation to ensure that barriers of communication in the clinics are destroyed as these barriers might lead to poor patient outcome.

### Recommendations

Negative workplace relationships between the nurse managers and union leaders affect the performance of the mining company and therefore should be prevented at all costs by resolving conflicts amicably. Union leaders and primary healthcare nurse managers should perceive each other as team members and not as enemies or opponents. Positive workplace relationships should be encouraged and be built continuously to promote teamwork, which will result in the achievement of collective goals within the mining industry. A study exploring and describing the perceptions of union leaders, nurses and doctors on workplace relationships in a mining industry is recommended as it will clarify how they perceive this phenomenon from their side.

### Limitations

There was limited data on mining primary healthcare; hence it was difficult for the researcher to support this context with literature from the same context. This study is discussing the perceptions of nurse managers working in the mining industry only; it will be of significance to extend it to nurse managers working in private and public primary healthcare clinics as well.

## Conclusion

Nurse managers in this study had both negative and positive perceptions on workplace relationships. Their negative perceptions were on union leaders; they perceived union leaders as autocratic, challenging and difficult to work with, and this resulted in negative workplace relationships between these two parties. The positive perceptions of nurse managers were on positive workplace relationships among the nurses and doctors at the clinics. Building positive workplace relationships was stressed as a critical obligation that the nurse managers and union leaders should work on collectively in this study.
